# Femorotibial angle scan–rescan reproducibility: A high‐precision calculation on a large cohort

**DOI:** 10.1002/ksa.12352

**Published:** 2024-07-10

**Authors:** Thomas A. G. Hall, Gareth G. Jones, Richard J. van Arkel

**Affiliations:** ^1^ Department of Mechanical Engineering Biomechanics Group, Imperial College London London UK; ^2^ MSk Lab, Department of Surgery and Cancer Imperial College London London UK

**Keywords:** alignment, biomechanics, osteoarthritis, radiography

## Abstract

**Purpose:**

Femorotibial angle (FTA) is a convenient measure of coronal knee alignment that can be extracted from a short knee radiograph, avoiding the additional radiation exposure and specialist equipment required for full‐leg radiographs. While intra‐ and inter‐reader reproducibility from the same image has been reported, the full scan–rescan reproducibility across images, as calculated in this study, has not.

**Methods:**

In this study, 4589 FTA measurement pairs from 2586 subjects acquired a year apart were used to estimate FTA scan–rescan reproducibility using data from the Osteoarthritis Initiative. Subjects with radiographic progression of osteoarthritis or other conditions that may cause a change in coronal knee alignment were excluded. Measurement pairs were analysed using paired‐samples t tests to detect differences and compared to symptomatic changes in Western Ontario and McMaster Universities Arthritis Index scores for joint pain, stiffness and physical function to detect correlations.

**Results:**

The 95% limit of agreement and the paired‐samples correlation were calculated with high precision to be [−1.76°, +1.78°] and 0.938, considerably worse than the corresponding figures for intra‐ and inter‐reader reproducibility, without relation to symptomatic or radiographic changes in knee condition. This error will weakly attenuate $R^{2}$ and r values from their true values in correlative studies involving FTA. The realistic maximum value for $R^{2}$ is 87% and for Pearson's r is 93%.

**Conclusion:**

The scan–rescan reproducibility in FTA is almost double the intra‐ and inter‐reader reliability from a single scan. At almost ±2° accuracy, FTA is inappropriate for surgical use, but it is sufficiently reproducible to produce good correlations in studies predicting disease incidence and progression.

**Level of Evidence:**

Level II, retrospective study.

**Abbreviations**
FTA: femorotibial angle
HKA: hip–knee–ankle angle
KL: Kellgren–Lawrence System for Classification of Osteoarthritis
OAI: Osteoarthritis Initiative
TKR: total knee replacement
WOMAC: Western Ontario and McMaster Universities Arthritis Index

## INTRODUCTION

Coronal knee alignment is a key consideration in the clinical management of knee osteoarthritis [5], a degenerative disease that affects 16% of the global population [4] and the planning of knee replacement and high tibial osteotomy surgeries. Alignment is measured using either femorotibial angle (FTA) or hip–knee–ankle (HKA) angle, where FTA is an anatomical angle that can be assessed on a short knee radiograph and HKA is a mechanical angle that requires a full‐limb radiograph [11]. While HKA is the gold‐standard measure, full‐limb radiographs require specialist equipment and expose patients to considerably more radiation (0.15–0.51 vs. 0.03 mSv) [14]. Hence, there has been persistent interest in producing accurate measurements of alignment from short knee radiographs [10, 21, 22, 26, 28], which includes the recent application of automated and artificially intelligent algorithms [7, 16, 31]. FTA also has utility in predicting the incidence and progression of knee osteoarthritis [2, 6, 12, 13, 15, 20, 24, 27, 29].

When setting performance expectations for predicting FTA or using it as a predictor, it is necessary to fully understand the inherent variability in this measurement across both acquisition and interpretation. While several FTA measurement approaches have been proposed [8, 14, 30], reproducibility has exclusively been evaluated in terms of intra‐ and inter‐reader reproducibility from a single scan (interpretation) but not in terms of the full reproducibility from scan to scan (acquisition and interpretation), presumably because a study of this nature would double the radiation exposure for each subject. Indeed, the only studies of scan–rescan reproducibility of any measure of coronal knee alignment are two small studies of eight and 30 subjects [18, 19], which measured bilateral HKA twice sequentially to produce 16 and 60 measurement pairs, respectively. In the latter, a specific protocol for controlling patient positioning was introduced to ensure HKA reproducibility, so this may not reflect routine clinical practice. The aim of this study, therefore, was to address this gap in the literature by providing a high‐precision calculation for the scan–rescan reproducibility of FTA without exposing patients to a double dose of radiation.

## MATERIALS AND METHODS

### Source data set

**Figure 1:**
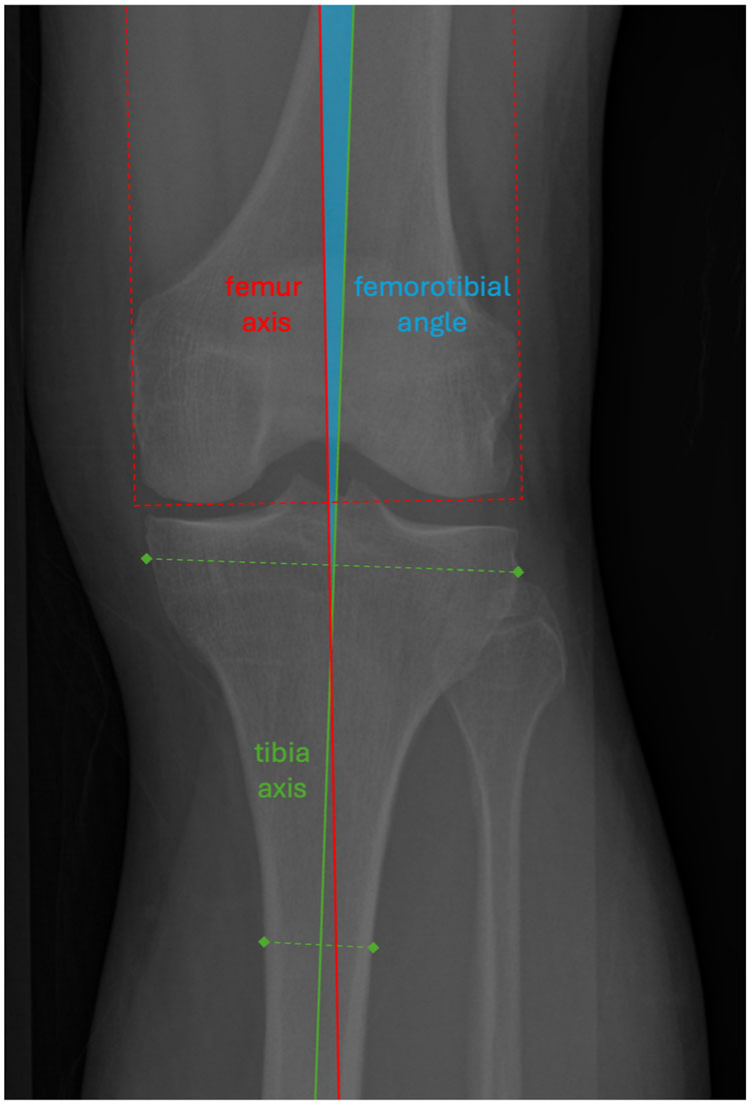
Radiograph showing the axes of the femur (red) and the tibia (green) for measurement of femorotibial angle (blue). The femur axis is perpendicular to a line tangent to the base of the femoral condyles and centred between the outer margins of the medial and lateral femoral epicondyles. The tibia axis is the line connecting the midpoints between the outer margins of the tibia at 1 and 10 cm below the lowest point on the tibial plateau. Varus alignments are negative.

All information for this study was drawn from the Osteoarthritis Initiative (OAI), a multicentre, longitudinal, prospective, observational study of knee osteoarthritis. Enrolment criteria for the original OAI study are available from the permanent archive hosted by the National Institutes of Health [17]. Differences in within‐subject FTA measurements from a baseline visit and a 12‐month follow‐up were used to estimate the scan–rescan reproducibility. FTA measurements were sourced from bilateral posteroanterior fixed‐flexion knee radiographs, which had been acquired using a protocol [23] where knees were flexed to 20–30° and feet were internally rotated to 10°. Each FTA measurement had been calculated using the semiautomatic method of Iranpour‐Boroujeni et al. (Figure 1) [8]. In all, there were 5753 FTA measurement pairs from 3218 subjects. Positive angles indicated valgus alignment. Western Ontario and McMaster Universities Osteoarthritis Index (WOMAC) scores were matched by side and timepoint to FTAs, providing corresponding patient‐recorded outcomes for pain, stiffness and function for each measurement. Additional information used to calculate data set characteristics and enact exclusion criteria was drawn from the Enrollees Demographics, Clinical Information, X‐ray Outcomes and Knee X‐ray Semiquantitative Scoring subsets.

### Exclusion criteria

**Figure 2:**
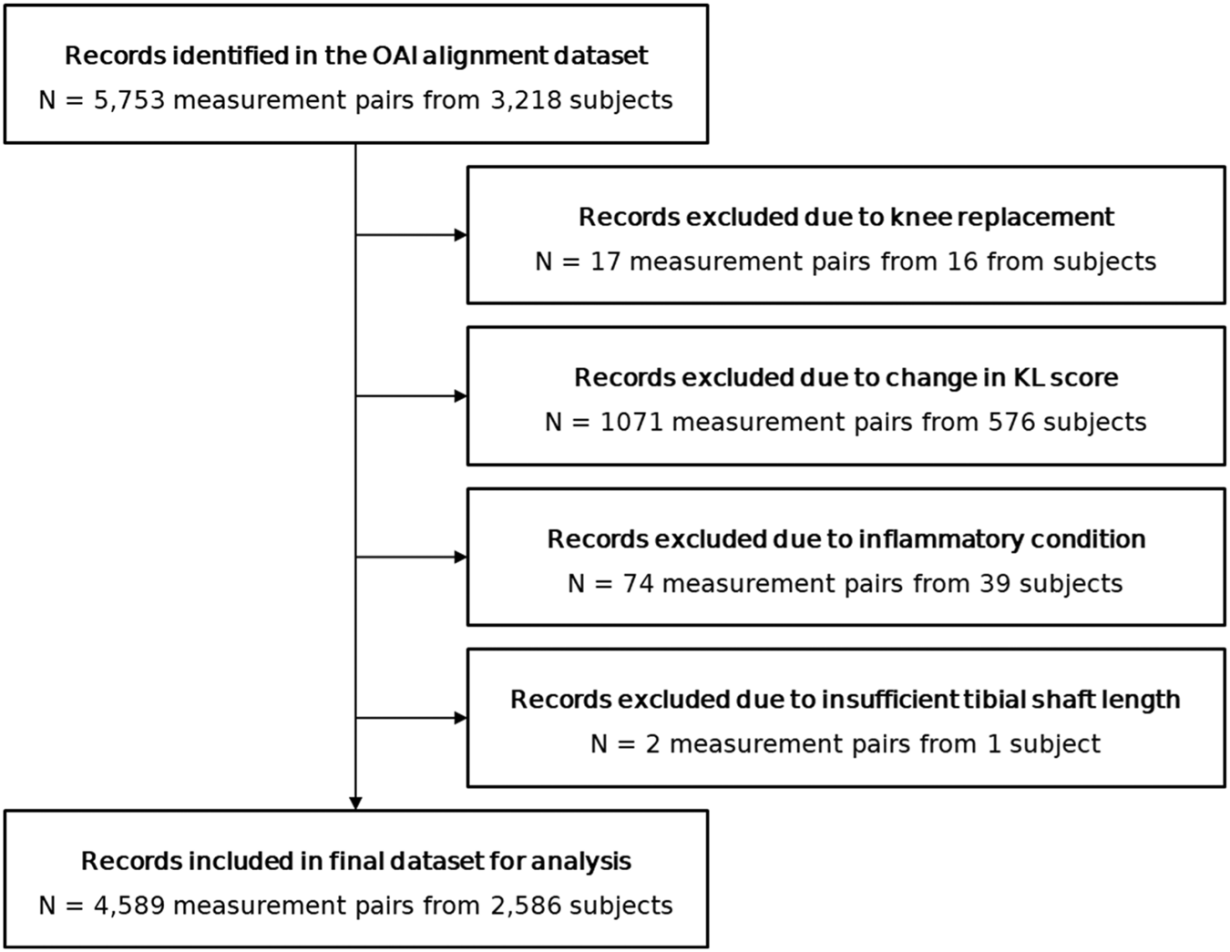
Preparation of the data set from the Osteoarthritis Initiative (OAI) X‐ray alignment data. No subjects were excluded due to a stroke, cerebrovascular accident, a blood clot or bleeding on the brain or a transient ischaemic attack.

Five criteria, which may cause a sudden and permanent change in coronal knee alignment or prevent its measurement, were set a priori to exclude study subjects from the analysis (Figure 2):

(1) Subjects that received a knee replacement (ipsilateral or contralateral) between the baseline visit and the 12‐month follow‐up.
(2) Subjects that exhibited observed changes in the radiographic appearance of their knee as measured by Kellgren–Lawrence (KL) grade from the baseline visit to the 12‐month follow‐up.
(3) Subjects with rheumatoid arthritis or polymyalgia rheumatica and inflammatory musculoskeletal disorders, which may cause transient changes in coronal knee alignment.
(4) Subjects that experienced a stroke, a cerebrovascular accident, a blood clot or bleeding in the brain or a transient ischaemic attack between the baseline visit and the 12‐month follow‐up.
(5) Subjects whose radiographs have less than 10 cm of tibia visible.

### Statistical analysis

Mean differences with 95% confidence intervals (CIs), standard deviations of the difference and Cohen's effect sizes were calculated for the repeated measures of FTA and WOMAC scores. Kolmogorov–Smirnov and Shapiro–Wilk tests were used to test the normality assumption of the within‐subject differences required for paired‐samples t tests and correlations.

The paired‐samples t tests were used to determine whether there were any statistically significant differences between repeated measures. Pearson's correlation coefficients between FTA and the WOMAC scores were calculated to determine the amount of variance that could be explained by changes in patient‐reported symptoms for pain, stiffness and physical function. With 4604 measurement pairs, the respective tests were sensitive to effect sizes of |d| > 0.012 and correlations of |r| > 0.053 ($R^{2}$ > 0.003) with 95% confidence and 95% power.

Paired‐sample correlation coefficients and 95% limits of agreement between the baseline visit and 12‐month follow‐up were reported as measures of FTA scan–rescan reproducibility. The effects of FTA scan–rescan reproducibility on coefficients of determination $R^{2}$ and Pearson's correlation coefficients r were calculated using an additive error model [25]:

(1) $$\frac{R_{\mathrm{observed}}^{2}}{R_{\mathrm{true}}^{2}}=\frac{\sigma_{\mathrm{true}}^{2}}{\sigma_{\mathrm{observed}}^{2}}=\frac{\sigma_{\mathrm{observed}}^{2}-\sigma_{\mathrm{diff}}^{2}}{\sigma_{\mathrm{observed}}^{2}},$$

(2) $$\frac{r_{\mathrm{observed}}}{r_{\mathrm{true}}}=\frac{\sigma_{\mathrm{true}}}{\sigma_{\mathrm{observed}}}=\sqrt{\frac{R_{\mathrm{observed}}^{2}}{R_{\mathrm{true}}^{2}}},$$

where $\sigma_{\mathrm{observed}}$ was the standard deviation of the FTA at baseline and $\sigma_{\mathrm{diff}}$ was the standard deviation of the difference FTA between the baseline and the 12‐month follow‐up.

The statistical analysis was performed using IBM SPSS Statistics software.

## RESULTS

### Data set composition

**Figure 3:**
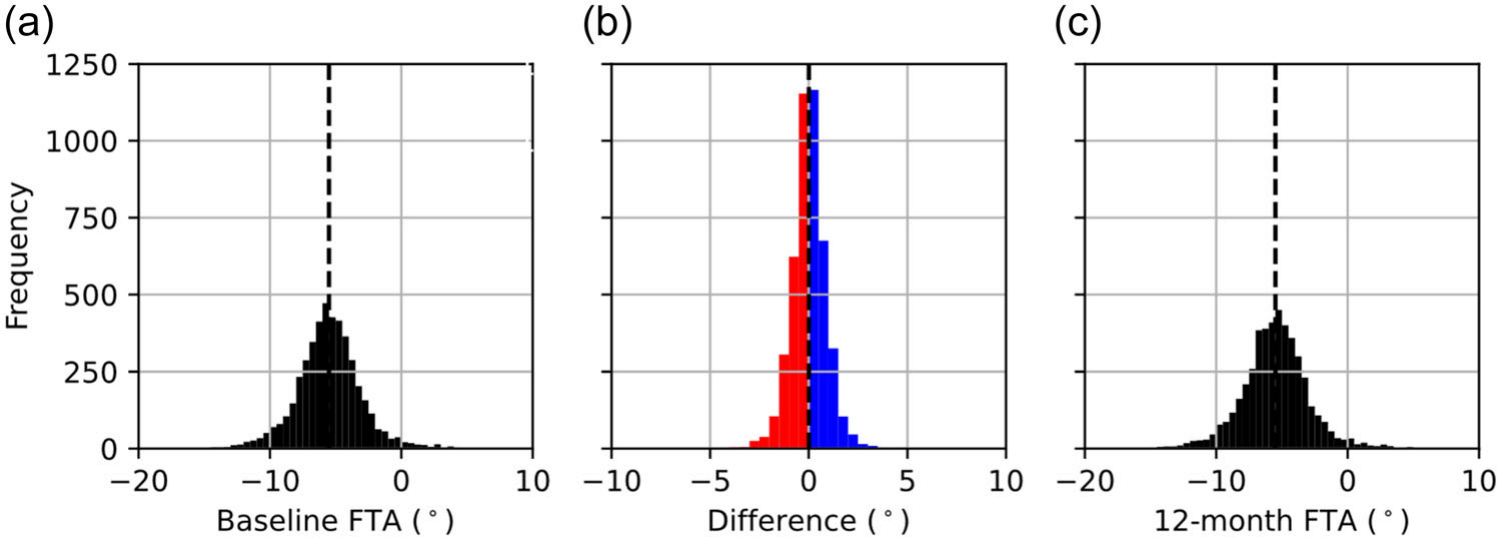
Distribution of femorotibial angle (FTA) at the baseline visit (a) and the 12‐month follow‐up (c) and the distribution of within‐subject FTA differences (b). Mean measurements are shown as dashed black lines.

**Figure 4:**
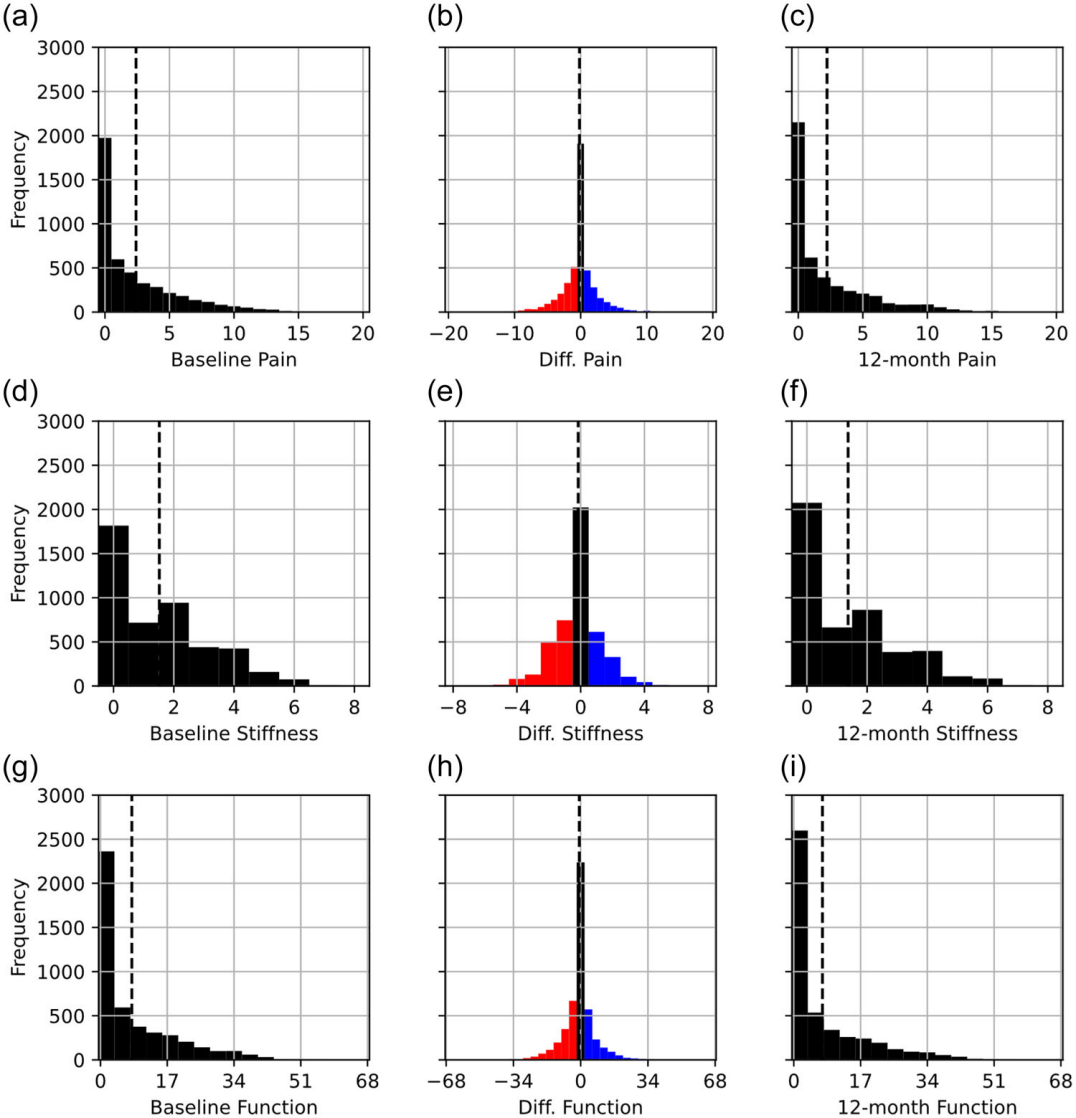
Distributions in Western Ontario and McMaster Universities Arthritis Index scores for pain (top: a–c), stiffness (middle: d–f) and function (bottom: g, h, i) across the maximum possible range at the baseline visit (left: a, d, g) and the 12‐month follow‐up (right: c, f, i) and the distribution of within‐subject differences (centre: b, e, h).

On application of exclusion criteria (Figure 2), the final data set consisted of 4589 FTA measurements from 2586 subjects, two measurements were sourced from 2003 subjects and one image was sourced from 583 subjects. The data set consisted of 1108 male subjects (42.8%) and 1478 female subjects (57.2%). The mean age was 61.6 years, with a standard deviation of ±9.2 years and a range of 45–79 years. The mean and standard deviation in FTA were –5.51° and ±2.52° at the baseline visit and –5.50° and ±2.59° at the 12‐month follow‐up visit (Figure 3). The mean score for baseline pain was 2.41 out of 20; the mean score for baseline stiffness was 1.52 out of 8; and the mean score for physical function was 7.98 out of 68 (Figure 4). The mean time between images was 416 days (1 year and 51 days) with a standard deviation of ±40 days.

### Within‐subject differences and correlations

The within‐subject differences in WOMAC scores and FTA measurements between the baseline visit and the 12‐month follow‐up visit were all near‐normal (Table 1). There were statistically significant differences (all p < 0.001) with small effect sizes (d) in all WOMAC scores between visits (Table 2; all p < 0.001): pain decreased by 0.18 points (normalised out of 100: 0.9; effect size: d = –0.07); stiffness decreased by 0.15 points (normalised: 1.9; d = –0.11) and physical function decreased by 0.69 points (normalised: 1.01; d = –0.08). The 95% limits of agreement normalised out of 100 for each WOMAC parameter were [–26.2, +28.0] for pain, [–37.3, +33.4] for stiffness and [–22.7, +24.8] for physical function.

**Table 1:** Tests indicating the near‐normality of the change in WOMAC scores and FTA measurement between the baseline visit and the 12‐month follow‐up.

	Kolmogorov–Smirnov	Shapiro–Wilk
Femorotibial angle	D (4589) = 0.049	W (4589) = 0.975
WOMAC: Pain	D (4589) = 0.209	W (4589) = 0.879
WOMAC: Stiffness	D (4589) = 0.223	W (4589) = 0.923
WOMAC: Function	D (4589) = 0.158	W (4589) = 0.883

*Note*: Both statistics vary from 0 to 1, but the Kolmogorov–Smirnov *D* statistic represents maximum difference with respect to the normal distribution (*D* = 0 is the optimum), while a Shapiro–Wilk *W* statistic value closer to 1 indicates closer alignment.

Abbreviations: FTA, femorotibial angle; WOMAC, Western Ontario and McMaster Universities Arthritis Index.

**Table 2:** Paired‐samples t test results for differences in FTA measurements and WOMAC scores (*italics*: normalised out of 100) between the baseline visit and the 12‐month follow‐up.

	Mean [95% CI]	Standard deviation	t Statistic	Sig.	Effect size
Femorotibial angle	+0.01° [–0.02°, +0.04°]	σ = 0.90°	t(4588) = +0.82	p = 0.410	d = +0.01
WOMAC: Pain	–0.18 [–0.26, –0.10] *–0.91 [–1.32, –0.51]*	σ = 2.76 σ = *13.8*	t(4588) = –4.48	p < 0.001	d = –0.07
WOMAC: Stiffness	–0.15 [–0.20, –0.11] *–1.92 [–2.44, –1.40]*	σ = 1.44 σ = *18.0*	t(4588) = –7.21	p < 0.001	d = –0.11
WOMAC: Function	–0.69 [–0.93, –0.46] *–1.02 [–1.37, –0.67]*	σ = 8.24 σ = *12.1*	t(4588) = –5.70	p < 0.001	d = –0.09

Abbreviations: CI, confidence interval; FTA, femorotibial angle; WOMAC, Western Ontario and McMaster Universities Arthritis Index.

**Figure 5:**
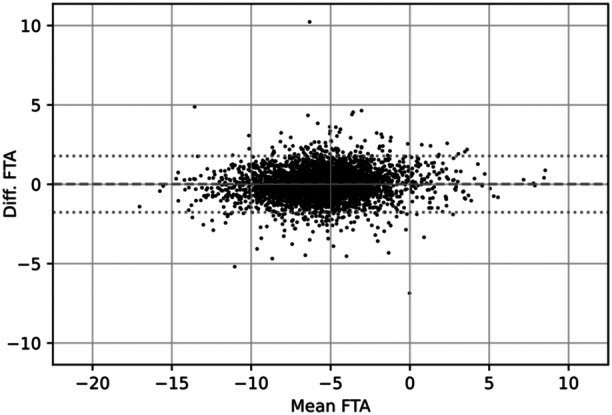
Bland–Altman plot showing the paired‐samples differences in femorotibial angle (FTA) (y axis) versus paired‐sample averages (x axis). The dashed line in each image indicates the mean difference (0.09°; p = 0.455) and the dotted line indicates the 95% limits of agreement ([−1.761, +1.781]).

No mean within‐subject difference in FTA between the baseline measurement and the 12‐month follow‐up was detected (Figure 5; difference: +0.01°; 95% CI: [−0.02°, +0.04°]; p = 0.415). No correlations between the changes in FTA and changes in WOMAC scores were detected (Table 3): 95% CIs for Pearson's correlation coefficients greater than r were all within the interval [−0.027, +0.055].

**Table 3:** Correlations between differences in FTA measurements and differences in WOMAC scores between the baseline visit and the 12‐month follow‐up.

	Correlation [95% CI]	Significance	$R^{2}$ Value
WOMAC: Pain	r = +0.026 [–0.002, +0.055]	p = 0.073	$R^{2}$ < 0.001
WOMAC: Stiffness	r = +0.001 [–0.027, +0.030]	p = 0.924	$R^{2}$ < 0.001
WOMAC: Function	r = +0.006 [–0.023, +0.035]	p = 0.687	$R^{2}\;$< 0.001

Abbreviations: CI, confidence interval; FTA, femorotibial angle; WOMAC, Western Ontario and McMaster Universities Arthritis Index.

### Scan–rescan reproducibility

The 95% limits of agreement for FTA scans 12 months apart were [−1.76°, +1.78°], with a 95% confidence on each limit of ±0.03° (Figure 5). The paired‐samples correlation between the baseline measurement and the 12‐month follow‐up was 0.938 (p < 0.001; 95% CI: [0.934, 0.941]). The standard deviation of the difference was ±0.90°—a ratio of 0.36 to the standard deviation of the baseline FTA ( ± 2.52°). The corresponding effects on *R* ^2^ and *r* values for correlations involving FTA were the reduction of the true values to 87% and 93%, respectively.

## DISCUSSION

This study addresses a gap in the literature for the scan–rescan reproducibility of FTA measurement: the 95% limit of agreement (LoA) and paired‐samples correlation of FTA measurements taken approximately one year apart were [−1.76°, +1.78°] and 0.938. Both figures were calculated with high precision due to the large data set of 4589 paired‐samples measurements from 2586 subjects. Capturing full scan‐to‐scan variability, these figures are notably worse than the intra‐ and inter‐reader reproducibility (both 95% LoA: ±1°; both correlation: 0.98) reported for FTA measurement from a single scan [8].

This analysis was based on a large patient cohort, which had shown no radiographic evidence of osteoarthritic knee joint deterioration or improvement. There were statistically significant within‐subject differences in patient‐reported outcome measures (WOMAC scores), but these effects on sizes (max: 0.11) were smaller than the minimum clinically important differences for pain (0.91 vs. 11), stiffness (1.92 vs. 8) and physical function (1.02 vs. 9; all normalised out of 100) [3]. Further, changes in WOMAC scores were uncorrelated with changes in FTA, and even at the extremes of the 95% confidence limits, changes in WOMAC scores explained less than 1% of the variance in FTA.

Compared to the only other studies of scan–rescan reproducibility for coronal knee alignment, the results were similar to a study of 8 subjects that reported a variation of ±2° [19] but higher than a study of 30 subjects (60 images) that reported 95% limits of agreement of [−1.13°, +1.15°] [18]. In the second study, full‐leg radiographs were acquired 1 week apart, and subject position was specifically controlled for a new measurement protocol under test. The relative contributions of the difference in rescan time, the difference in target (HKA vs. FTA) and the non‐routine measurement protocol to the disparity cannot be apportioned in this analysis.

At almost ±2° in scan–rescan reproducibility, FTA is already at the margins of acceptable accuracy for joint replacement surgery, ±3° historically [9] and ±2° in an era of personalised surgery [1], irrespective of any femoral bowing outside of the short knee radiograph. FTA is, therefore, unsuitable as a measure for planning surgical procedures. However, with the scan–rescan reproducibility attenuating $R^{2}$ and r values to theoretical maxima of 87% and 93% of their true values, respectively, it is still possible to achieve good correlations when using FTA as an independent variable, such as in studies of osteoarthritis incidence and progression. While the true strength of relationships involving anatomical alignment will be underestimated, the ‘noise’ introduced by this measurement error will only weakly mask the ‘signals’ of true relationships. There will also be a performance‐limiting effect on algorithms predicting multi‐image trends in FTA progression or out‐of‐image anatomical measures, such as HKA [7, 21, 31].

As a limitation, the data set for this analysis did not include any knees with a knee replacement. However, provided the patient has the same implant across images, and that the bearing has not considerably worn between timepoints, it is improbable that the presence of an implant will affect scan–rescan reproducibility. The results are also uncontrolled for weight changes and the development of any neuromuscular disorders, which both have the potential to cause some variation in coronal knee alignment but were not included within the original data set. The analysis also only considered FTA measured using the method of Iranpour‐Boroujeni et al. [8]. Other methods for FTA measurement exist [14, 30] and may produce different reproducibility results.

## CONCLUSION

FTA scan–rescan reproducibility is almost double the inter‐reader reproducibility reported from a single scan (almost ±2° vs. ±1°), and this increased variability could not be explained by any change in the radiographic appearance, pain, stiffness or physical function of the subjects' knees. While unsuitable for surgical planning, FTA is a reproducible measurement for correlative studies, with realistic maximum $R^{2}$ and r values of 87% and 93%, respectively.

## AUTHOR CONTRIBUTIONS

**Thomas A. G. Hall**: Conceptualisation; methodology; investigation; software; data curation; visualisation; writing—original draft. **Gareth G. Jones**: Methodology, writing—review and editing. **Richard J. van Arkel**: Methodology; writing—review and editing.

## CONFLICT OF INTEREST STATEMENT

The authors declare no conflict of interest.

## ETHICS STATEMENT

The data in this study were sourced from an Osteoarthritis Initiative (OAI) public‐use data set. Patients gave informed consent to participate in the original OAI study, which was overseen by an independent Observational Study Monitoring Board appointed by the National Institute of Arthritis and Musculoskeletal and Skin Diseases (NIAMS) at the National Institutes of Health (NIH). No specific ethical approval was required for this study.

## Data Availability

Source data is available from the National Institute of Mental Health Data Archive (NDA) at nda.nih.gov/oai.
